# Supplementary material for: Predicting visual function by interpreting a neuronal wiring diagram
Source: Nature. 2024 Oct 2;634(8032):113–23. doi: 10.1038/s41586-024-07953-5 (PMC11446822; doi:10.1038/s41586-024-07953-5)
Supplement: Supplementary file 6 — CRF and ERF predictions for individual TmY4 and TmY9 cells. Analogous to Supplementary Data 3, but for TmY target types. Shown are the top four monosynaptic pathways, the strongest pathway passing through each of the top ten intermediary types (ranking from Extended Data Fig. 7), and the trisynaptic pathway Tm1–TmY–Dm3–TmY (see the section entitled Prediction of spatial normalization). [file 41586_2024_7953_MOESM6_ESM.zip › DataS4/TmY9q/720575940628540932.pdf]

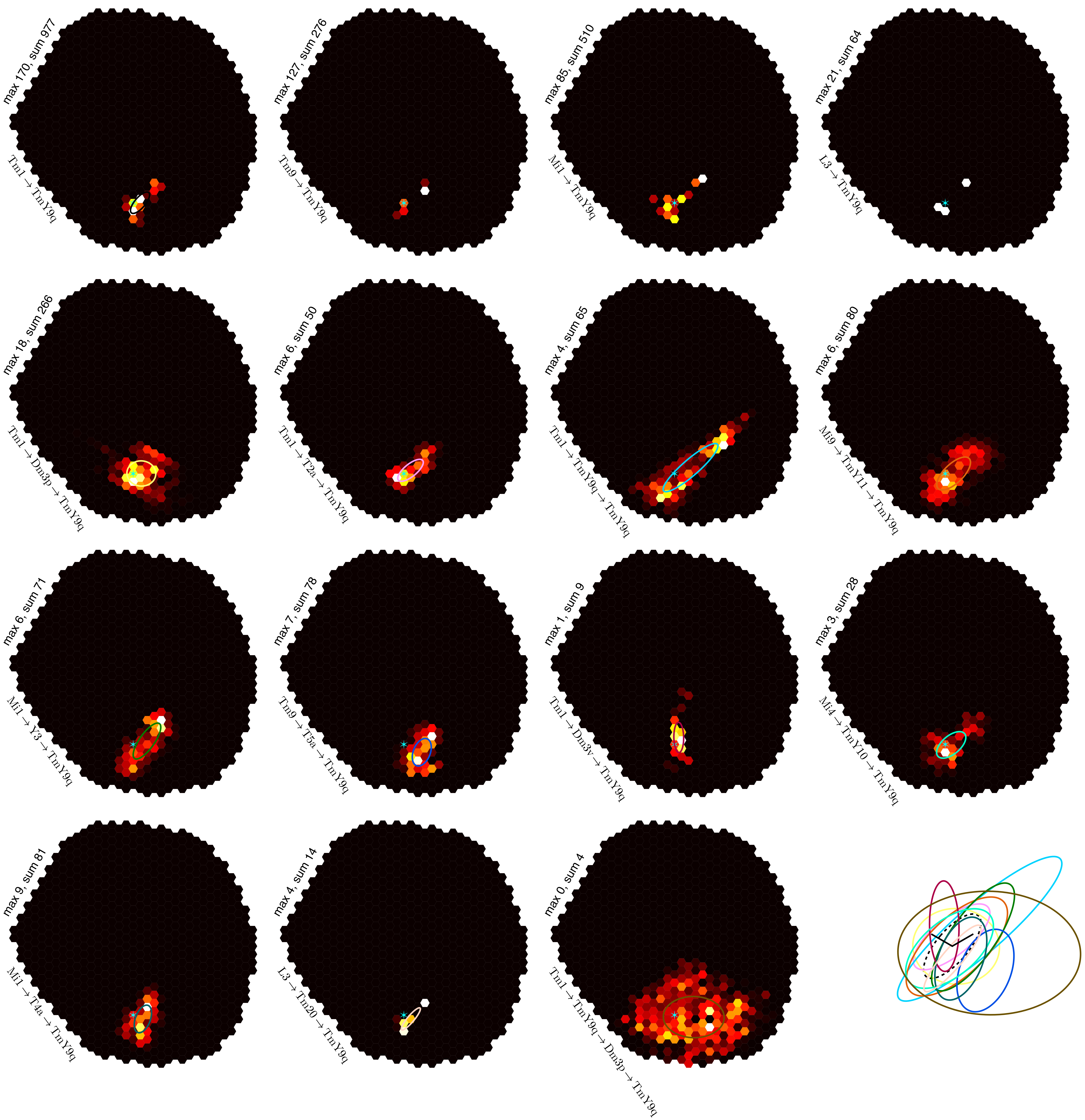
max 170, sum 977
Tm1 → TmY9q
max 127, sum 276
Tm9 → TmY9q
max 85, sum 510
Mi1 → TmY9q
max 21, sum 64
L3 → TmY9q
max 18, sum 266
Tm1 → Dm3p → TmY9q
max 6, sum 50
Tm1 → T2a → TmY9q
max 4, sum 65
Tm1 → TmY9q → TmY9q
max 6, sum 80
Mi9 → TmY11 → TmY9q
max 6, sum 71
Mi1 → Y3 → TmY9q
max 7, sum 78
Tm9 → T5a → TmY9q
max 1, sum 9
Tm1 → Dm3v → TmY9q
max 3, sum 28
Mi4 → TmY10 → TmY9q
max 9, sum 81
Mi1 → T4a → TmY9q
max 4, sum 14
L3 → Tm20 → TmY9q
max 0, sum 4
Tm1 → TmY9q → Dm3p → TmY9q
